# Effects of Dietary Supplementation of Omega-3 PUFA Enriched Fish Oil During Late-Pregnancy and Lactation on Reproductive Performance, Immune Activity and Fecal Microbiota Composition in Postpartum Sows

**DOI:** 10.3390/vetsci12020139

**Published:** 2025-02-07

**Authors:** Zihao Ge, Yalong An, Wei Lan, Xiao Li

**Affiliations:** 1 Anhui Engineering Research Center for Functional Fruit Drink and Ecological Fermentation, Anhui Province Key Laboratory of Embryo Development and Reproductive Regulation, School of Biological and Food Engineering, Fuyang Normal University, Fuyang 236037, China; 2 Key Laboratory of Animal Genetics and Breeding in Shaanxi Province, College of Animal Sciences and Technologies, Northwest A&F University, Xianyang 712100, China

**Keywords:** fish oil, post-weaning estrus, sow, lactation, gestation

## Abstract

Delayed or failed postpartum estrus can impede the reproductive performance of rebreeding dams. Our study aimed to test whether dietary fish oil can promote estrus return and improve fecal microbiota in multiparous sows. Forty-five sows were randomly allocated into three treatments: a basal diet, or a basal diet plus 30 or 60 g/day of fish oil from gestation day 90 to lactation day 21. Results showed that dietary fish oil significantly elevated circulating DHA in sows, without significant changes in litter size, litter weight, or backfat loss. Supplementation of fish oil (30 or 60 g/day) shortened the interval from weaning to estrus by 1.8 days and 1.67 days, respectively, associated with a significant boost of estradiol. Increases in prolactin and IgA were only significant in the high-dosage group. In addition, plasma MDA and antioxidant activities were up-regulated by fish oil, combined with elevated serum IL-1β and IL-6. Moreover, dietary fish oil significantly reduced serum zonulin, improved the Simpson index of fecal microbiota, and increased the abundance of *Lactobacillus* and *Ruminococcaceae_UCG-014* genera. In conclusion, dietary omega-3 PUFA-enriched fish oil provides a promising approach to aiding estrus return and reshaping fecal microbiota in post-weaning sows.

## Simple Summary

Postpartum estrus is crucial for the reproductive performance of dams, significantly influencing the productivity and efficiency of the livestock industry. This study addressed the issue of delayed or failed postpartum estrus in sows and investigated the effects of dietary fish oil on enhancing their reproductive performance. Our findings demonstrated that fish oil, as a dietary intervention, can shorten the interval from weaning to estrus, enhance immune activity, and reshape the fecal microbiota, thereby improving the overall reproductive health of sows. In conclusion, this study suggests that dietary fish oil supplementation is an effective strategy to facilitate estrus return and modulate fecal microbiota in multiparous sows, ultimately improving the productivity of pig farms.

## 1. Introduction

The reproductive cycle of female mammals is precisely orchestrated by endocrinal hormones, the levels of which are largely influenced by environmental factors including dietary ingredients [1]. In the livestock industry, dams, especially sows, dairy cows, and goats are subjected to repeated breeding to ensure continuous production of meat or milk and to maximize reproductive efficiency. A timely and relatively fixed weaning-to-estrus interval (WEI, around 5–7 days in sows) is a decisive factor in subsequent ovulation, artificial insemination, and embryo survival [2]. However, approximately 15% of sows fail to return to estrus within 7 days [3]. The delayed or failure to return to an estrus cycle in postpartum sows could result in extra costs in the swine industry, and a similar situation occurs in dairy farms [4].

Emerging evidence has indicated that peripartum supplementation of omega-3 polyunsaturated fatty acids (ω-3 PUFAs) could benefit gestational outcomes in humans [5]. Moreover, the administration of fish oil, which is a rich source of ω-3 PUFAs, has been found to be beneficial for utero-ovarian functions in repeat breeding of dairy cows [6], ewes [7] and goats [8]. Similarly, the intake of ω-3 PUFAs has been observed to reduce the WEI by approximately 1.2 days in cyclic sows [9]; this suggests that fish oil supplementation can have a positive effect on the reproductive cycle of sows, although the underlying pathways remain unknown.

Fecal samples are frequently utilized as a substitute for gut microbiota, because they are easily obtained and provide a reasonable representation of the gut microbial community [10,11]. Recent reports have highlighted the importance of gut microbiota in various biological processes including reproduction [12,13]. A newly published report has revealed that dysbiosis of the fecal microbiome is strongly associated with anestrus in young gilts [14] and weaned sows [15]. Dietary supplementation of isomaltooligosaccharide or *Bacillus* has been found to reduce the WEI of sows by altering the structure of fecal microbiome [16]. The fecal microbial community is directly or indirectly influenced by dietary ω-3 PUFAs and is involved in mediating the beneficial functions of ω-3 PUFAs in various physiological and pathological states [17,18]. Thus, we hypothesized that ω-3 PUFAs enriched fish oil could shorten post-weaning estrus and improve fecal microbial flora in postpartum sows.

## 2. Materials and Methods

### 2.1. Experimental Design and Animal Care

Animal management and sampling procedures were approved by the Animal Care and Use Committee of Fuyang Normal University, China.

In this study, low (30 g/d/sow) or high (60 g/d/sow) dosages of fish oil (containing 22% of ω-3 PUFAs; the ratio of eicosapentaenoic acid (EPA) to docosahexaenoic acid (DHA) was 1:2.2) were included in the sow diet during late gestation and lactation. The reproductive performance, oxidative and immune status, and fecal microbiota were recorded at weaned sows (21 days after farrowing). Correlation analysis was performed to further examine the interplay between fecal microbiota and serum hormones.

Forty-five sows with similar gestational dates (Landrace × Yorkshire, 85 days of gestation, parity = 1, first-time pregnancy, body weight 212.35 ± 1.61 kg, mean ± SE), reared at the Hengzhuang pig farm (Hanzhong, Shaanxi, China), were randomly allocated into three diet groups: a basal diet group and a basal diet plus 30 or 60 g/day of fish oil group. Each dietary treatment included 15 replicates, with one sow per replicate. During each feeding session, one-third of the total feed was initially provided, with fish oil evenly sprinkled over it. Once the sows had consumed this portion, the remaining two-thirds of the feed was added. The fish oil was purchased from Baishilu BioTech Co. Ltd. (Guangzhou, China). The fatty acid composition of fish oil was determined by GC-MS and the concentrations of EPA and DHA are 5.9 and 13.3 mg/mL, respectively (as shown in Table A2). After a pre-trial of 5 days, the experiment commenced on day 90 of gestation and concluded on day 21 of lactation. On day 108 of gestation, sows were switched into lactation feed. The sow diets during late gestation and lactation were supplied by Power Feed (Xi’an, Shaanxi, China), with the feed ingredients detailed in Table A1. Prior to farrowing, sows were fed twice a day (08:00 a.m. and 15:00 p.m.) with a total of 3 kg feed. Parturitions were unassisted to prevent stress from human intervention. During parturition, each sow received 1 kg of feed, with successive increases of 0.5 kg per day. Two weeks later, sows were fed ad libitum via an automated feeder, ensuring a constant feed supply and enabling them to eat at will. Sows were individually housed in farrowing stalls (2.1 m × 0.65 m) until gestational day 110 and were transferred to 2.2 m × 1.8 m concrete-floored delivery pens (Hanzhong, Shaanxi, China) throughout lactation. Throughout the feeding period, sows had free access to water and were vaccinated in accordance with the company’s standard procedures.

### 2.2. Data Recording

Throughout the experimental period, the daily feed intake of the sows was meticulously recorded, and the average daily feed intake was subsequently calculated. The total litter size, surviving litter size, number of stillborns, and litter birth weight were documented within 24 h of farrowing. The backfat thickness at P2 (at the last rib) of sows was measured using a Rego Lean-meater (Hanzhong, Shaanxi, China) on day 110 of gestation and day 21 of lactation. During the measurement, enough coupling agent was applied on the measurement site to ensure full contact between the probe and the skin, the probe was held vertically and flat against the pig’s skin surface, and the instrument reading was then recorded. The piglets were weaned at 21 d of age, after which the sows were transferred to the breeding facility. The lactation capacity of sows was measured by litter weight gain of piglets at 21 days of age (litter weight gain = litter weight at weaning minus litter weight alive at parturition), as previously described [19]. The lactation volume of sows is estimated to be 4 kg milk for each 1 kg gain of piglets, and lactation volume of sows = litter weight gain × 4 [20].

### 2.3. Estrus Identification

After weaning, estrus detection was performed twice a day with a boar present until estrus was detected. Estrus detection was performed from weaning until sows showed the standing reflex to the back-pressure test and the swelling and reddening of the vulva. And the interval from weaning to first estrus was recorded.

### 2.4. Sample Collection

On the weaning day (day 21 of lactation), eight sows were randomly selected from each group, and blood samples were collected in heparinized tubes from the vena jugularis, ensuring minimal stress to the sows. The blood samples were then incubated at 4 °C for 10–15 min, and centrifuged at 4 °C for 15 min at 1200× *g* to obtain clear serum for further analysis. On the same day, fresh fecal samples of the sows were collected individually using 20 mL centrifuge tubes and stored at −80 °C for subsequent 16S rRNA sequencing. The heparinized tubes and 20 mL centrifuge tubes were purchased from Yangling Sanli Chemical Glass Supply Station (Yangling, Shaanxi, China).

### 2.5. Hormonal Assay

Serum biochemical indicators were assessed using a Beckman Coulter AU5800 biochemical analyzer (Yangling, Shaanxi, China). Levels of reproductive hormones (progesterone, oxytocin, estradiol E2, prolactin), immunoglobulins (IgA, IgG, and IgM), inflammatory factors (IL-1β, IL-6, TNF-α, IFN-γ, and IL-10), intestinal permeability index (zonulin), as well as growth factors (T3, T4, IGF1, insulin, and cortisol) were measured with commercial kits from Shanghai Hengyuan Biological (Shanghai, China, as listed in Table A3). The OD values were measured using a Thermo FC Microplate reader (Shanghai, China). Serum activities of total antioxidant (T-AOC), superoxide dismutase (SOD), glutathione peroxidase (GSH-Px), and catalase (CAT) and the content of malondialdehyde (MDA) were measured using commercial kits purchased from Nanjing Jiancheng Bioengineering Institute (Nanjing, Jiangsu, China, also listed in Table A3), in accordance with the manufactures’ instructions.

### 2.6. 16S rRNA Sequencing

Microbial DNA was extracted from fecal samples using a DNeasy PowerSoil kit (Qiagen, Hilden, Germany). The V3–V4 hypervariable regions within the 16S rRNA sequence were cloned with universal primers (338F-806R), and the amplicons were purified and screened on an Illumina HiSeq™ 2500 platform, with the help of Biomarker Technologies (Beijing, China). Raw sequences were assembled using the QIIME and FLASH packages, and effective fragments were obtained using UPARSE and clustered into operational taxonomic units (OTUs) using UCLUST at a 97% sequence identity threshold. Subsequently, high-quality sequences were compared against the Ribosomal Database Project classifier program to assign taxonomy (v.2.20) based on a 90% confidence threshold. Alpha-diversity analysis via the Shannon index as well as the abundance-based coverage (ACE), Chao1, and Simpson indices was performed using the Mothur software package (ver. 1.32.0). The bacterial taxonomic differences represented between groups at phylum, family, and genus levels were analyzed using LEfSe (Line Discriminant Analysis Effect Size) [21].

### 2.7. Statistical Analysis

Statistical analyses were performed using SPSS 20.0 software (SPSS Inc., Chicago, IL, USA). Data were firstly subjected to the Shapiro–Wilk test to check whether they were normally distributed, followed by Levene to test the homogeneity of variances, and then data were processed with one-way ANOVA and LSD tests. The results were considered significant at a probability level of *p* ≤ 0.05, and probability values between 0.05 and 0.10 were considered a trend. Spearman correlation analysis was performed to examine the interplay between special fecal microbiota and serum bio-indicators, followed by Bonferroni correction of the results. Graphical representations in bar graph format were performed through GraphPad Prism software v.10.4.1 (GraphPad Software, Inc., La Jolla, CA, USA).

## 3. Results

### 3.1. Dietary Administration of Fish Oil Shortened the Weaning-Estrous Interval in Sows

Dietary supplementation of fish oil led to an increasing tendency of circulating ω-3 PUFAs. Notably, DHA increased significantly in a dose-dependent manner, while EPA showed an upward tendency without reaching statistical significance. In contrast, α-linolenic acid (ALA), another important member of the ω-3 PUFA family, remained unaltered following dietary administration of fish oil. The ratio of ω-6 PUFA to ω-3 PUFA was significantly decreased, mainly due to the elevated DHA (Table 1). Furthermore, the effects of dietary fish oil on other fatty acids in the serum of sows are shown in Table A4. Caproate (C6:0), caprylate (C8:0), petroselaidate (C18:1N12T), transvaccenat (C18:1N7T), linoelaidate (C18:2N6T), 7-transnonadecenoate (C19:1N12T), trans 11-Eicosenoate (C20:1T), heneicosanoate (C21:0), brassidate (C22:1N9T), erucate (C22:1N9), and docosadienoate (C22:2) in the serum presented a reducing tendency. Unndecanoate (C11:0), myristelaidate (C14:1T), 10-transpentadecenoate (C15:1T), 10-transsheptadecenoate (C17:1T), and nervonoate (C24:1) decreased as the dosage of fish oil. 10-Transnonadecenoate (C19:1N9T) in the serum showed an increasing tendency.

The total number born, number of born alive, litter weight alive at parturition, average weight of piglets born alive, duration of farrowing, lactation capacity, and lactation volume were not significantly affected by dietary administration of fish oil (Table 2). Notably, the WEI was significantly shortened by dietary intake of fish oil (Table 2). Furthermore, the body weight, food intake, and backfat thickness of sows supplemented with fish oil were comparable with those in the control group throughout late pregnancy and lactation (Table A5).

### 3.2. Dietary Administration of Fish Oil-Altered Estrus-Related Factors in Lactating Sows

Considering that the heat cycle appeared to commence earlier in sows fed with fish oil, we further examined whether the circulating levels of estrus-related hormones or factors were affected. The results showed that serum estradiol and prolactin in lactating sows were significantly increased by dietary supplementation of fish oil. The circulating progesterone and oxytocin levels, however, did not exhibit significant changes in response to fish oil treatment. Regarding other endocrine factors, serum IGF1, T3, and T4 were not significantly altered by the dietary administration of fish oil, whereas cortisol was significantly reduced by the low-dose treatment compared to the high-dose treatment (Table 3).

### 3.3. Dietary Administration of Fish Oil Enhanced Immune Activity and Antioxidant Capacity in Lactating Sows

In the present study, serum IgA was significantly increased by 26% exposure to the high dose of fish oil, while IgG and IgM levels did not exhibit significant changes in response to fish oil supplementation (Table 4).

Regarding serum antioxidants, the serum MDA content as well as CAT and SOD activities were markedly elevated by dietary administration of fish oil. Serum T-AOC activity in lactating sows was not significantly changed by dietary fish oil administration, and plasma GSH-Px activity tended to be increased by intake of fish oil, particularly in the low-dose group (Table 5).

With respect to inflammatory markers, serum IL-1β in lactating sows was induced by dietary administration of fish oil in the 60 g/d group, while circulating IL-6 was increased by dietary administration of fish oil, regardless of the dosage. The serum IFN-γ, TNF-α, IL-10, and C-reactive protein (CRP) were not significantly affected by dietary administration of fish oil (Table 5). Furthermore, dietary fish oil supplementation had no significant effects on other serum biochemical indexes (Table A6).

### 3.4. Dietary Administration of Fish Oil Improved Fecal Health in Lactating Sows

**Figure 1:**
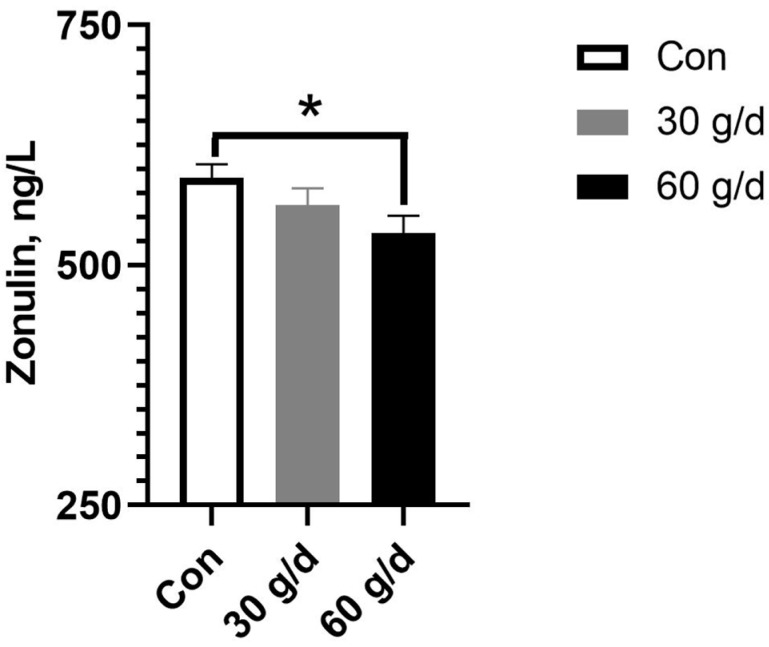
Effects of fish oil treatment during late gestation and lactation on the concentration of plasma zonulin in sows. * means significantly different between groups (*p* < 0.05, *n* = 8).

**Figure 2:**
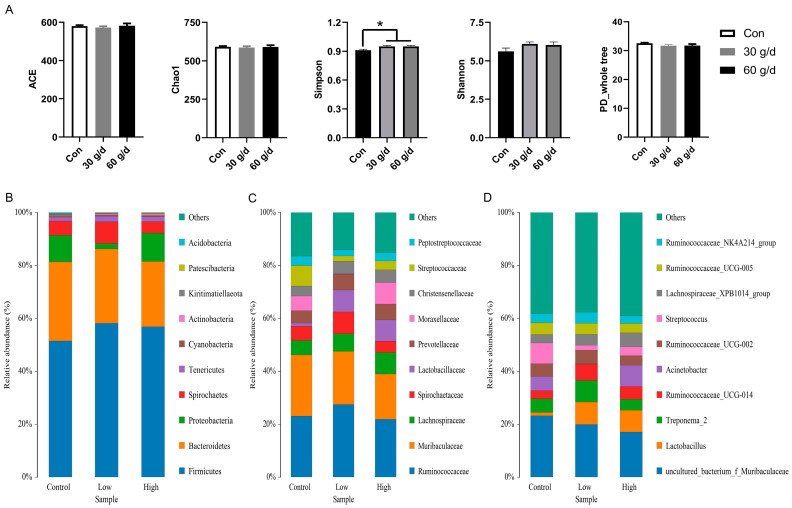
Effects of fish oil treatment during late gestation and lactation on fecal microbiome in sows. (**A**) Effects of fish oil treatment on fecal microbial diversity indices. (**B**) Composition of fecal microbiota at the phylum level. (**C**) Composition of fecal microbiota at the family level. (**D**) Composition of fecal microbiota at the genus level. * means significantly differently between groups (*p* < 0.05, *n* = 8).

The circulating concentration of zonulin, which serves as an indicator of intestinal permeability, was significantly decreased by dietary administration of fish oil (Figure 1), suggesting that fish oil enhances intestinal health. A lot of evidence has shown that the gut microbiota is an important target of dietary administration of fish oil [17,18], and thus, the composition of the gut microbiota was assessed using fresh fecal samples. The 16S rRNA sequencing revealed that the Simpson index was significantly increased by dietary administration of fish oil, indicating that intake of fish oil facilitated the diversity of the gut microbiota (Figure 2A). Moreover, fish oil enriched the abundance of the phylum Actinobacteria (Figure 2B) and significantly enriched the family Lactobacillaceae (Figure 2C) as well as the genus *Lactobacillus*. Fish oil also increased the enrichment of the genus *Ruminococcaceae_UCG-014* and the effect was much more effective in the low-dose treatment (Figure 2D).

Furthermore, Spearman correlation analysis was performed to detect the association between serum biomarkers and fecal microbiota. However, the results were not significant after the Bonferroni correction.

## 4. Discussion

Omega-3 PUFAs have been linked to human health throughout all stages of life, from fetal development to aging, and the beneficial effects of dietary fish oil on pregnancy and breastfeeding have been extensively documented [5]. In our study, dietary administration of fish oil significantly elevated circulating ω-3 PUFAs in sows (Table 1), and had negligible effects on the number of piglets born per litter, or the numbers born alive and stillborns (Table 2), in line with previous studies [9,22]. Higher infantile birth weight induced by maternal fish oil was once reported in human [23,24]; however, increased litter size and number born alive were only observed in primiparous sows, indicating a strong interaction between parity and marine ω-3 PUFAs [25].

In our study, supplementation of fish oil exerted no significant effects on food intake, body weight loss, or backfat loss in lactating sows (Table A5), suggesting that dietary fish oil did not significantly affect energy status in sows. This could be attributed to the fact that unlike short- and medium-chain fatty acids, which serve as immediate sources of energy, ω-3 PUFAs primarily function as essential components of biological membranes, precursors to various endocrine factors, or are secreted into milk [5,26].

In the modern swine industry, a relatively fixed WEI is extremely necessary for batch production [27], and an extended WEI is associated with reduced pregnancy rates and embryonic survival [28], while shorter WEI co-occurs with a higher farrowing rate in the subsequent cycle [29]. In our study, dietary administration of fish oil led to a significant reduction in the WEI, in accordance with a previous study in multiparous sows [9]. Similarly, dietary intake of fish oil during late gestation also resulted in the early occurrence of behavioral estrus in Rohilkhandi goats [8].

Earlier post-weaning estrus in sows fed with fish oil may be attributed to the elevated serum estradiol (Table 3). Estradiol is a critical hormone modulating estrus cycle in pigs [30]. The concentration of serum estradiol was very low in sows with anestrus after weaning [31], and lower estradiol plasma levels are associated with impairments in fertility in adult rats [32]. A previous report demonstrated that fish oil could restore the decreased estradiol and ovarian function in streptozotocin-diabetic rats [33]. Furthermore, dietary supplementation of fish oil resulted in a significant boost of serum IgA concentration, with slight increases in IgG and IgM, while significant increases in IgA, IgG, and IgM were detected in the plasma of sows fed with ω-3 PUFAs during the periparturient period [9]. Dietary supplementation with DHA plus EPA during late gestation (60 days prior to expected foaling dates) and early lactation also resulted in an increase in IgG (T) in focal serum [34]. The increased levels of circulating immunoglobulins induced by dietary fish oil are supposed to ensure good health, which could benefit estrous return in lactating sows.

Intriguingly, incorporating fish oil in the diet significantly elevated serum MDA content as well as SOD and GSH-Px capacities in lactating sows in our work, which is also observed in previous studies where fish oil increased the susceptibility to oxidative stress in sows [35], and the elevated MDA might be due to the oxidized ω-3 PUFAs. In addition, plasma IL-1β and IL-6 were largely elevated in lactating sows by dietary intake of fish oil in the current study, which may be caused by the increase in MDA production [36] instead of bacterial infection, as plasma CRP in lactating sows was not significantly influenced by fish oil intake (Table 5). Moreover, supplementary fish oil here significantly boosted plasma zonulin (Figure 1), a biomarker of intestinal permeability [37], which is considered a major pathway for the well-documented anti-inflammatory functions of fish oil [26]. Although the minimal effects of fish oil on oxidative stress in sows [38] were also reported, the microcapsules of fish oil or supplementation combined with antioxidants are still preferred in future use of fish oil [39].

Gut microbiota are emerging as a vital factor determining post-weaning estrus in sows [15], and differs greatly among normal-return and anestrous sows [40,41]. The composition of gut microbiota is directly or indirectly influenced by ω-3 PUFA status in multiple ways [17]. In the present study, dietary administration of fish oil significantly increased the Simpson index of fecal microbiota in lactating sows, indicating that the diversity of the gut microbiota was enhanced by fish oil in accordance with traditional understanding [17], and a previous report with a similar experimental design in sows [9]. Furthermore, the structure of fecal microbiota in lactating sows was also significantly altered by dietary intake of fish oil, as the abundance of the phylum *Actinobacteria*, the family *Lactobacillaceae*, and the genus *Ruminococcaceae_UCG-014* and *Lactobacillus* was remarkably boosted by fish oil.

Notably, the abundance of *Ruminococcaceae_UCG-014*, one of the main butyrate-producing bacteria [42], was also elevated following dietary intake of fish oil, and dietary supplementations with sodium butyrate during late pregnancy and lactation led to shorter WEl of sows [9]. Mechanically, butyric acid could up-regulate estradiol secretion in porcine granulosa cells, as an ex vivo experiment revealed [43]. Combined with *Lactobacillus*, the abundance of *Lactobacillus,* a well-known probiotic for both human and livestock animals [44], was increased in weaning sows treated with fish oil in the present study. Increased enrichment of *Lactobacillus* was also detected in normal estrus return of weaning sows, compared with the non-return sows [15]. Thus, we propose gut microbiota might serve as one of important links between dietary fish oil, boost of estradiol, and improved utero-ovarian functions in lactating sows, although the exact pathway concerning how *Lactobacillus* works needs further investigation.

However, it is important to acknowledge some limitations in our research. Firstly, our study was limited to a group of parity 1 sows, but WEI is a factor that can impact sows across all parity levels. Therefore, further experiments are required to explore how fish oil affects WEI across different parities, providing a more comprehensive understanding of its effects throughout the sow’s reproductive history. Additionally, due to certain constraints, blood samples were collected only at weaning, which represents another limitation of our study. Collecting samples at multiple time points could show the dynamic changes in blood parameters during the experiment. This would enable us to determine whether the changes observed at weaning are the result of a gradual accumulation or if there are specific periods when the supplementation has a more pronounced effect.

## 5. Conclusions

Collectively, our study has revealed that dietary administration of fish oil is an effective approach to facilitating estrus return in lactating sows, associated with changes in the composition of fecal microbiota. The increased abundances of *Ruminococcaceae_UCG-014* and *Lactobacillus* by fish oil intake were positively correlated with the increases in plasma estradiol attributed to post-weaning estrus, although the causal relationship between dietary administration of fish oil and gut microbiota community deserves further study. In addition, it should be mentioned that proper dosage and combination with antioxidants are recommended when fish oil is further administered in pig farms.

## Figures and Tables

**Table 1:** Effects of fish oil treatment during late gestation and lactation on fatty acid composition in the serum of sows.

	Fish Oil, g per Day per Sow			SEM	*p*-Value
	0	30 g/d	60 g/d		
EPA, μg/mL	3.34	4.97	6.47	1.916	0.082
DHA, μg/mL	3.49 ^b^	6.63 ^ab^	10.51 ^a^	2.965	0.014
ALA, μg/mL	3.31	3.16	3.95	1.082	0.502
ω-3 PUFA, μg/mL	10.14	14.76	20.94	5.914	0.052
LA, μg/mL	69.52	64.69	81.68	34.772	0.736
GLA, μg/mL	2.51	2.29	2.05	0.780	0.660
AA, μg/mL	23.63	16.56	14.91	14.183	0.618
ω-6 PUFA, μg/mL	95.50	83.55	98.65	49.210	0.879
ω-6 PUFA/ω-3 PUFA	8.72 ^a^	5.75 ^b^	4.94 ^b^	1.328	0.004

Note: EPA = eicosapentaenoic acid (C20:5n3); DHA = docosahexaenoic acid (C22:6n3); ALA = α-linolenic acid (C18:3n3); PUFA = polyunsaturated fatty acid; LA = linoleic acid (C18:2n6); GLA = γ-linoleic acid (C18:3n6); AA = Arachidonic acid (C22:4n6). ^a,b^ Values with different letters in the same row are significantly different (*p* < 0.05); *n* = 8 per treatment.

**Table 2:** Effects of fish oil administration late gestation and lactation on the productive performance of sows.

	Fish Oil, g per Day per Sow			SEM	*p*-Value
	0	30 g/d	60 g/d		
No. born per litter	13.73	13.67	15.33	2.496	0.130
No. born alive	11.80	12.47	13.07	2.569	0.409
No. healthy piglets	10.67	11.53	11.60	2.303	0.471
No. weak piglets	1.13	0.93	1.47	1.095	0.411
No. stillborn	1.47	1.07	1.80	1.293	0.507
Litter weight alive at parturition, kg	15.31	16.70	17.16	3.948	0.419
Average weight of piglets born alive, kg	1.29	1.34	1.32	0.157	0.643
Litter weight at weaning, kg	73.72	80.53	81.58	10.581	0.110
Duration of farrowing, min	253.33	225.00	214.00	116.790	0.639
WEI, day	6.07 ^a^	4.27 ^b^	4.40 ^b^	1.486	0.003
Lactation capacity	58.44	63.98	65.45	8.711	0.121
Lactation volume, kg	233.76	255.92	261.80	34.842	0.121

Note: Lactation capacity was defined as the litter weight gain during lactation, litter weight gain during lactation = litter weight at weaning-litter weight alive at parturition [19]. Lactation volume = litter weight gain during lactation × 4 [20]. ^a,b^ Values with different letters in the same row are significantly different (*p* < 0.05); *n* = 15 per treatment.

**Table 3:** Effects of fish oil during late gestation and lactation on endocrine reproductive factors in lactating sows.

	Fish Oil, g per Day per Sow			SEM	*p*-Value
	0	30 g/d	60 g/d		
Estradiol E2, pmol/L	123.24 ^b^	142.15 ^a^	142.95 ^a^	13.10	0.018
Prolactin, ng/L	54.23 ^b^	61.52 ^b^	75.24 ^a^	6.84	0.001
Progesterone, pmol/L	1699.94	1593.37	1658.56	175.34	0.530
Oxytocin, ng/L	46.40	47.89	48.07	5.32	0.817
IGF1, μg/L	9.54	9.86	10.15	1.05	0.671
Insulin, mIU/L	55.64	50.30	51.06	4.19	0.059
T3, pmol/L	99.20	102.38	109.41	13.08	0.473
T4, pmol/L	560.32	484.03	563.71	56.84	0.080
Cortisol, μg/L	117.69 ^a^	88.84 ^b^	108.65 ^a^	13.26	0.014

Note: IGF1 = insulin-like growth factor. ^a,b^ Values with different letters in the same row are significantly different (*p* < 0.05); *n* = 8 per treatment.

**Table 4:** Effects of fish oil administration during late pregnancy and lactation on circulating immunoglobulins content in lactating sows.

	Fish Oil, g per Day per Sow			SEM	*p*-Value
	0	30 g/d	60 g/d		
IgA, μg/mL	13.48 ^b^	14.67 ^ab^	16.98 ^a^	1.84	0.032
IgG, μg/mL	184.57	208.05	215.57	22.95	0.125
IgM, μg/mL	17.11	18.05	19.05	1.62	0.205

Note: ^a,b^ Values with different letters in the same row are significantly different (*p* < 0.05); *n* = 8 per treatment.

**Table 5:** Effects of fish oil administration during late pregnancy and lactation on serum antioxidant activity and inflammatory factors in lactating sows.

	Fish Oil, g per Day per Sow			SEM	*p*-Value
	0	30 g/d	60 g/d		
T-AOC, mmol/L	0.23	0.31	0.34	0.10	0.147
MDA, nmol/mL	4.38 ^b^	7.44 ^a^	7.79 ^a^	1.63	0.002
SOD, U/mL	106.21 ^b^	157.10 ^a^	157.65 ^a^	25.02	0.001
GSH-Px, U/mL	388.42 ^b^	423.45 ^a^	395.88 ^ab^	25.38	0.045
CAT, U/mL	1.95 ^b^	3.02 ^b^	5.64 ^a^	1.65	0.002
IL-1β, ng/L	16.10 ^b^	19.34 ^ab^	21.14 ^a^	2.36	0.017
IL-6, ng/L	1056.37 ^b^	1181.68 ^a^	1182.50 ^a^	75.06	0.032
IFN-γ, pg/mL	1169.69	1187.18	1299.31	158.49	0.402
TNF-α, pg/mL	167.54	170.03	188.18	20.42	0.163
IL-10, ng/L	102.32	96.18	105.23	18.83	0.745
CRP,/L	2287.75	2126.03	2221.05	161.83	0.200

Note: T-AOC = total antioxidant capacity; MDA = malondialdehyde; SOD = superoxide dismutase; GSH-Px = glutathione peroxidase; CAT = catalase; IL-1β = Interleukin 1 beta; IL-6 = Interleukin 6; IFN-γ = Interferon gamma; TNF-α = tumor necrosis factor alpha; IL-10 = Interleukin 10; CRP = C-reactive protein; ^a,b^ Values with different letters in the same row are significantly different (*p* < 0.05); *n* = 8 per treatment.

## Data Availability

All data generated or used during the study are available from the corresponding author upon reasonable request.

## Appendix A

**Table A1:** Feed ingredients and nutrition level of sow diet during late gestation and lactation.

Item	Gestation Diet	Lactation Diet
Corn (%)	48.80	63.20
46 Soybean meal (%)	-	20.00
43 Soybean meal (%)	12.70	-
Wheat flour (%)	-	5.00
Wheat bran (%)	20.00	3.00
Beet pulp (%)	8.00	-
Alfalfa meal (%)	4.00	-
Oil (%)	1.00	3.00
Yeast culture (%)	1.00	-
Import fish powder (%)	-	2.00
CaHPO4 (%)	1.30	1.20
Mineral feed (%)	0.80	1.20
Salt (%)	0.40	0.40
Premix (%)	2.00	1.00
Digestible energy (MJ/kg)	12.77	13.82
Crude protein (%)	16.49	18.39
Coarse ash (%)	5.49	5.08
Calcium (%)	0.99	0.62
Total phosphorus (%)	0.64	0.58
Lysine (%)	0.6	1.1
Methionine (%)	0.19	0.27
Threonine (%)	0.48	0.64

Note: Crude protein, coarse ash, calcium, and total phosphorus are the measured values; the rest are calculated values. Premix in late gestation contains medicinal stone 1.32%, methionine 0.05%, 98% lysine 0.15%, threonine 0.03%, valine 0.02%, phytase 0.04, multi-mineral 0.20%, multi-vitamin 0.03%, vitamin E 0.02%, antibiotic peptide 0.03%, edulcorator 0.04%, and 15% magnesium sulfate 0.08%; premix in lactation contains methionine 0.05%, 98% lysine 0.60%, valine 0.02%, phytase 0.04, multi-mineral 0.20%, multi-vitamin 0.03%, vitamin E 0.03%, and antibiotic peptide 0.04%.

**Table A2:** Fatty acid composition of fish oil used in current study.

Name	Abbreviation	The Concentration (μg/mL)
Eicosapentaenoate (EPA)	C20:5N3	5936.289
Docosahexaenoate (DHA)	C22:6N3	13,333.422
Caproate	C6:0	27.627
Caprylate	C8:0	31.908
Caprate	C10:0	23.095
Unndecanoate	C11:0	31.923
Laurate	C12:0	133.506
Tridecanoate	C13:0	74.947
Myristate	C14:0	3575.214
Myristelaidate	C14:1T	74.888
Myristoleate	C14:1	31.249
Pentadecanoate	C15:0	642.742
10-Transpentadecenoate	C15:1T	30.084
10-Pentadecenoate	C15:1	28.121
Palmitate	C16:0	14,792.208
Palmitelaidate	C16:1T	288.805
Palmitoleate	C16:1	4494.199
Heptadecanoate	C17:0	693.754
10-Transsheptadecenoate	C17:1T	116.88
10-Heptadecenoate	C17:1	305.8
Stearate	C18:0	3929.074
Petroselaidate	C18:1N12T	47.085
Elaidate	C18:1N9T	32.811
Transvaccenate	C18:1N7T	207.986
Petroselinate	C18:1N12	599.501
Oleate	C18:1N9C	11,906.25
Vaccenate	C18:1N7	2023.044
Linoelaidate	C18:2N6T	34.346
7-Transnonadecenoate	C19:1N12T	85.128
10-Transnonadecenoate	C19:1N9T	42.827
Linoleate	C18:2N6	6162.907
Arachidate	C20:0	312.336
Gamma Linolenate	C18:3N6	153.646
Trans 11-Eicosenoate	C20:1T	34.057
11-Eicosenoate	C20:1	792.849
Alpha Linolenate	C18:3N3	1502.661
Heneicosanoate	C21:0	92.092
11-14 Eicosadienoate	C20:2	207.826
Behenate	C22:0	172.247
Homogamma Linolenate	C20:3N6	116.971
Brassidate	C22:1N9T	25.023
Erucate	C22:1N9	1078.639
11-14-17 Eicosatrienoate	C20:3N3	470.27
Arachidonate	C20:4N6	1031.33
Tricosanoate	C23:0	103.727
Docosadienoate	C22:2	43.381
Lignocerate	C24:0	131.36
Nervonoate	C24:1	255.125
Docosatetraenoate	C22:4	160.603
Docosapentaenoate	C22:5N6	873.062
Docosapentaenoate	C22:5N3	1049.842

**Table A3:** Precision values of the ELISA kits employed for the quantitative measurement of serum biochemical indicators and antioxidant activity in serum from sows fed control or 30 or 60 g/day of fish oil group.

	%CV	
	Intra-Assay	Inter-Assay
Pig PROG ELISA Kit (HS034-Pg; Shanghai Hengyuan Biological, Shanghai, China)	<4.2%	<6.3%
Pig OT ELISA Kit (HS304-Pg; Shanghai Hengyuan Biological, Shanghai, China)	<4.3%	<6.7%
Pig E2 ELISA Kit (HS316-Pg; Shanghai Hengyuan Biological, Shanghai, China)	<5.7%	<8.9%
Pig PRL ELISA Kit (HS303-Pg; Shanghai Hengyuan Biological, Shanghai, China)	<6.7%	<8.5%
Pig IgA ELISA Kit (HS175-Pg; Shanghai Hengyuan Biological, Shanghai, China)	<5.2%	<7.3%
Pig IgG ELISA Kit (HS173-Pg; Shanghai Hengyuan Biological, Shanghai, China)	<4.9%	<8.5%
Pig IgM ELISA Kit (HS170-Pg; Shanghai Hengyuan Biological, Shanghai, China)	<5.4%	<7.3%
Pig IL-1β ELISA Kit (HS350-Pg; Shanghai Hengyuan Biological, Shanghai, China)	<5.9%	<8.2%
Pig IL-6 ELISA Kit (HS343-Pg; Shanghai Hengyuan Biological, Shanghai, China)	<5.3%	<8.7%
Pig IL-10 ELISA Kit (HS355-Pg; Shanghai Hengyuan Biological, Shanghai, China)	<6.1%	<8.9%
Pig zonulin ELISA Kit (HS427-Pg; Shanghai Hengyuan Biological, Shanghai, China)	<5.8%	<8.3%
Pig TNF-α ELISA Kit (HS015-Pg; Shanghai Hengyuan Biological, Shanghai, China)	<5.1%	<7.8%
Pig IFN-γ ELISA Kit (HS359-Pg; Shanghai Hengyuan Biological, Shanghai, China)	<4.3%	<9.5%
Pig T3 ELISA Kit (HS139-Pg; Shanghai Hengyuan Biological, Shanghai, China)	<5.3%	<7.2%
Pig T4 ELISA Kit (HS419-Pg; Shanghai Hengyuan Biological, Shanghai, China)	<6.1%	<7.8%
Pig IGF1 ELISA Kit (HS052-Pg; Shanghai Hengyuan Biological, Shanghai, China)	<6.8%	<9.6%
Pig INS ELISA Kit (HS057-Pg; Shanghai Hengyuan Biological, Shanghai, China)	<6.7%	<8.3%
Pig Cortisol ELISA Kit (HS157-Pg; Shanghai Hengyuan Biological, Shanghai, China)	<6.3%	<7.8%
Pig T-AOC ELISA Kit (A015; Nanjing Jiancheng Bioengineering Institute, Nanjing, Jiangsu, China)	<6.83%	<3.2%
Pig SOD ELISA Kit (A001; Nanjing Jiancheng Bioengineering Institute, Nanjing, Jiangsu, China)	<3.52%	<1.7%
Pig GSH-Px ELISA Kit (A005; Nanjing Jiancheng Bioengineering Institute, Nanjing, Jiangsu, China)	<4.34%	<3.1%
Pig CAT ELISA Kit (A007; Nanjing Jiancheng Bioengineering Institute, Nanjing, Jiangsu, China)	<4.94%	<1.9%
Pig MDA ELISA Kit (A003-1; Nanjing Jiancheng Bioengineering Institute, Nanjing, Jiangsu, China)	<4.11%	<3.5%

Abbreviations: PROG = progesterone; OT = oxytocin; E2 = estradiol E2; PRL = prolactin; IgA = Immunoglobulin A; IgG = Immunoglobulin G; IgM = Immunoglobulin M; IL-1β = Interleukin 1 beta; IL-6 = Interleukin 6; TNF-α = tumor necrosis factor alpha; IFN-γ = Interferon gamma; IL-10 = Interleukin 10; T3 = triiodothyronine; T4 = tetraiodothyronine; IGF1 = insulin like growth factor; INS = insulin; T-AOC = total antioxidant capacity; SOD = superoxide dismutase; GSH-Px = glutathione peroxidase; CAT = catalase; MDA = malondialdehyde.

**Table A4:** Effects of fish oil treatment during late gestation and lactation on other fatty acids in the serum of sows.

Name	Abbreviation	Fish Oil, g per Day per Sow			SEM	*p*-Value
		0	30 g/d	60 g/d		
Caproate	C6:0	0.09 ^a^	0.06 ^ab^	0.02 ^b^	0.029	0.022
Caprylate	C8:0	0.15 ^a^	0.13 ^b^	0.13 ^b^	0.008	0.025
Caprate	C10:0	0.14	0.11	0.12	0.022	0.221
Unndecanoate	C11:0	0.230 ^a^	0.23 ^ab^	0.22 ^b^	0.005	0.065
Laurate	C12:0	0.333	0.29	0.38	0.084	0.361
Tridecanoate	C13:0	0.59	0.57	0.57	0.016	0.263
Myristate	C14:0	2.54	2.40	3.63	0.880	0.151
Myristelaidate	C14:1 T	0.96 ^a^	0.92 ^ab^	0.76 ^b^	0.125	0.102
Pentadecanoate	C15:0	2.42	2.42	2.49	0.302	0.918
10-Transpentadecenoate	C15:1T	2.21 ^a^	1.96 ^ab^	1.86 ^b^	0.184	0.068
10-Pentadecenoate	C15:1	5.14	4.60	4.44	0.443	0.116
Palmitate	C16:0	152.64	139.68	160.26	38.689	0.756
Palmitelaidate	C16:1T	1.06	1.01	1.05	0.119	0.849
Palmitoleate	C16:1	6.30	4.62	5.66	2.926	0.723
Heptadecanoate	C17:0	5.32	5.34	4.68	1.155	0.663
10-Transsheptadecenoate	C17:1T	2.11 ^a^	1.96 ^ab^	1.84 ^b^	0.147	0.076
10-Heptadecenoate	C17:1	2.71	2.51	2.25	0.454	0.388
Stearate	C18:0	131.04	98.626	106.24	45.814	0.596
Petroselaidate	C18:1N12T	3.15 ^a^	2.47 ^ab^	1.99 ^b^	0.511	0.031
Elaidate	C18:1N9T	1.23	1.07	1.05	0.118	0.110
Transvaccenate	C18:1N7T	5.57 ^a^	4.85 ^ab^	4.55 ^b^	0.508	0.016
Petroselinate	C18:1N12	50.05	37.97	51.33	19.408	0.581
Oleate	C18:1N9C	87.10	58.95	62.83	44.684	0.642
Vaccenate	C18:1N7	7.99	5.99	7.13	3.447	0.722
Linoelaidate	C18:2N6T	0.96 ^a^	0.82 ^ab^	0.73 ^b^	0.114	0.050
7-Transnonadecenoate	C19:1N12T	1.54 ^a^	1.44 ^ab^	1.24 ^b^	0.138	0.034
10-Transnonadecenoate	C19:1N9T	2.59 ^a^	2.63 ^ab^	4.04 ^b^	0.620	0.014
Arachidate	C20:0	1.46	1.30	1.15	0.206	0.156
Trans 11-Eicosenoate	C20:1T	1.96 ^a^	1.74 ^ab^	1.50 ^b^	0.197	0.028
11-Eicosenoate	C20:1	2.48	2.21	2.52	0.517	0.669
Heneicosanoate	C21:0	1.12 ^a^	1.07 ^ab^	0.97 ^b^	0.052	0.009
11-14 Eicosadienoate	C20:2	2.29	2.15	2.02	0.364	0.614
Behenate	C22:0	0.69	0.66	0.64	0.067	0.518
Homogamma Linolenate	C20:3N6	2.39	2.15	1.95	0.621	0.622
Brassidate	C22:1N9T	1.75 ^a^	1.57 ^ab^	1.36 ^b^	0.195	0.055
Erucate	C22:1N9	3.02 ^a^	2.56 ^ab^	2.21 ^b^	0.320	0.018
11-14-17 Eicosatrienoate	C20:3N3	20.43	13.92	14.06	11.548	0.673
Tricosanoate	C23:0	1.63	1.62	1.61	0.019	0.155
Docosadienoate	C22:2	1.57 ^a^	1.45 ^ab^	1.24 ^b^	0.160	0.047
Lignocerate	C24:0	0.78	0.77	0.74	0.070	0.688
Nervonoate	C24:1	1.67 ^a^	1.55 ^ab^	1.37 ^b^	0.176	0.104
Docosatetraenoate	C22:4	2.29	1.64	1.41	0.634	0.181
Docosapentaenoate	C22:5N6	1.32	9.19	13.95	15.397	0.528
Docosapentaenoate	C22:5N3	5.72	5.08	4.01	2.827	0.700

Note: ^a,b^ Values with different letters in the same row are significantly different (*p* < 0.05); *n* = 8 per treatment.

**Table A5:** Effects of fish oil administration during late gestation and lactation on food intake, body weight, and backfat thickness of sows.

		Fish Oil, g per Day per Sow			SEM	*p*-Value
		0	30 g/d	60 g/d		
Body weight	Day 90 of gestation, kg	212.00	212.33	212.73	11.061	0.984
	Day 110 of gestation, kg	226.07	228.33	227.93	11.448	0.846
	Day 21 of lactation, kg	200.20	205.27	207.07	13.000	0.334
	Loss during lactation, mm	25.93	23.07	20.87	10.646	0.433
Feed intake, kg	Gestation	66.62	66.32	65.69	1.392	0.190
	Lactation	98.75	100.91	97.67	6.093	0.339
	Total (during late gestation and lactation)	165.37	167.24	163.36	6.460	0.270
Backfat thickness	Day 110 of gestation, mm	22.80	21.60	22.60	2.276	0.312
	Day 21 of lactation, mm	18.93	18.33	18.60	2.785	0.840
	Loss during lactation, mm	3.87	3.27	4.00	2.313	0.655
	Percentage of Loss during lactation, %	16.95	15.06	17.53	10.075	0.783

**Table A6:** Effects of dietary EPA and DHA on serum biochemical indices in lactating sows.

	EPA + DHA, % of Diet			SEM	*p*-Value
	0	0.2	0.4		
Glutamate transaminase, U/L	18.14	24.43	19.14	6.456	0.281
Glutathane transaminase, U/L	21.71	21.29	14.71	12.119	0.601
Alkaline phosphatase, U/L	27.43	35.14	30.14	9.580	0.450
Total protein, g/L	29.83	38.49	33.94	9.718	0.390
Albumin, g/L	13.40	17.83	15.59	4.545	0.328
Globulin, g/L	16.43	20.66	18.36	5.332	0.470
Albumin/Globulin	0.83	0.87	0.86	0.106	0.786
Lactate dehydrogenase, U/L	339.29	268.43	229.43	136.554	0.451
Creatine Kinase, U/L	1065.57	706.14	524.57	675.109	0.451
Urea, mmol/L	2.51	2.94	2.73	0.710	0.648
Creatinine, μmol/L	62.46	81.86	79.41	20.890	0.302
Glucose, mmol/L	1.78	2.47	2.24	0.606	0.215
Triglycerides, mmol/L	0.19	0.23	0.29	0.108	0.349
Total cholesterol, mmol/L	1.03	1.39	1.14	0.368	0.305
HDL-c, mmol/L	0.34	0.53	0.43	0.149	0.194
LDL-c, mmol/L	0.40	0.56	0.44	0.158	0.294
